# Key role for spinal dorsal horn microglial kinin B_1 _receptor in early diabetic pain neuropathy

**DOI:** 10.1186/1742-2094-7-36

**Published:** 2010-06-29

**Authors:** Sébastien Talbot, Emna Chahmi, Jenny Pena Dias, Réjean Couture

**Affiliations:** 1 Department of Physiology, Faculty of Medicine, Université de Montréal, C.P. 6128, Succursale Downtown, Montréal, Québec, H3C 3J7, Canada

## Abstract

**Background:**

The pro-nociceptive kinin B_1 _receptor (B_1_R) is upregulated on sensory C-fibres, astrocytes and microglia in the spinal cord of streptozotocin (STZ)-diabetic rat. This study aims at defining the role of microglial kinin B_1_R in diabetic pain neuropathy.

**Methods:**

Sprague-Dawley rats were made diabetic with STZ (65 mg/kg, i.p.), and 4 days later, two specific inhibitors of microglial cells (fluorocitrate, 1 nmol, i.t.; minocycline, 10 mg/kg, i.p.) were administered to assess the impact on thermal hyperalgesia, allodynia and mRNA expression (qRT-PCR) of B_1_R and pro-inflammatory markers. Spinal B_1_R binding sites ((^125^I)-HPP-desArg^10^-Hoe 140) were also measured by quantitative autoradiography. Inhibition of microglia was confirmed by confocal microscopy with the specific marker Iba-1. Effects of intrathecal and/or systemic administration of B_1_R agonist (des-Arg^9^-BK) and antagonists (SSR240612 and R-715) were measured on neuropathic pain manifestations.

**Results:**

STZ-diabetic rats displayed significant tactile and cold allodynia compared with control rats. Intrathecal or peripheral blockade of B_1_R or inhibition of microglia reversed time-dependently tactile and cold allodynia in diabetic rats without affecting basal values in control rats. Microglia inhibition also abolished thermal hyperalgesia and the enhanced allodynia induced by intrathecal des-Arg^9^-BK without affecting hyperglycemia in STZ rats. The enhanced mRNA expression (B_1_R, IL-1β, TNF-α, TRPV1) and Iba-1 immunoreactivity in the STZ spinal cord were normalized by fluorocitrate or minocycline, yet B_1_R binding sites were reduced by 38%.

**Conclusion:**

The upregulation of kinin B_1_R in spinal dorsal horn microglia by pro-inflammatory cytokines is proposed as a crucial mechanism in early pain neuropathy in STZ-diabetic rats.

## Background

According to the World Health Organization, over 300 millions of people worldwide will be diagnosed with diabetes mellitus by the year 2025. Diabetes leads to micro- and macro-vascular complications such as hypertension, retinopathy, nephropathy, sensory and autonomic polyneuropathies [1]. Patients with diabetic sensory neuropathy experience a variety of aberrant sensations including spontaneous pain, hyperalgesia and hypersensitivity to non-painful stimuli, which is commonly known as allodynia [2,3]. Epidemiological data demonstrated that peripheral diabetic polyneuropathy affects 50-60% of diabetic patients and nowadays is recognized as the most difficult pain to treat since it is largely resistant to commercially available treatments [3-5]. The lack of knowledge regarding the exact mechanism leading to diabetes-induced neuropathic pain put emphasis on the need to identify cellular and molecular targets to develop new therapeutic approaches.

Recent studies highlighted a primary role for the inducible kinin B_1 _receptor (B_1_R) in mediation of nociception and diabetes-induced neuropathic pain [6,7]. Kinins are defined as pro-inflammatory and vasoactive peptides, which act through the activation of two G-protein-coupled receptors (R) denoted as B_1 _and B_2 _[8,9]. The B_2_R is widely and constitutively expressed in central and peripheral tissues and is activated by its preferential agonists bradykinin (BK) and Lys-BK. The B_1_R is activated by the active metabolites des-Arg^9^-BK and Lys-des-Arg^9^-BK and has a low level of expression in healthy tissues [10]. The latter receptor is upregulated after exposure to pro-inflammatory cytokines, bacterial endotoxins, hyperglycemia-induced oxidative stress and diabetes [11-13]. B_1_R knockout mice are less sensitive to pro-inflammatory pain stimuli, spinal sensitization and diabetic hyperalgesia [14,15]. Pharmacological studies support a role for B_1_R in mechanical and/or thermal hyperalgesia induced by cytokines [16], formalin [17] and in neuropathic pain induced by peripheral nerve injury [18] or as consequence of type 1 and 2 diabetes mellitus [15,19-21]. Autoradiography studies showed a widespread distribution of kinin B_1_R binding sites in the spinal cord of diabetic rats [19,21-23]. This is consistent with the presence of B_1_R on neuronal and non-neuronal elements, including sensory C-fibres, astrocytes and microglia as revealed by confocal microscopy in the spinal cord of streptozotocin (STZ)-diabetic rats [22].

Microglia, known as macrophages of the central nervous system (CNS), have for primary function to phagocyte debris and other pathogens in the CNS [24]. Nevertheless, emerging evidence suggests an important role played by spinal microglial cells in STZ-induced pain neuropathy. For instance, microglial activation and the generation of neuropathies in STZ-diabetic rats were both prevented by Gabapentin treatment [25]. Moreover, spinal microglial cells are upregulated in neuropathic pain models of nerve injury [26,27]. Dorsal horn microglia activation is thought to play a pivotal role in diabetes-induced neuropathy via a MAPKp38α signaling pathway, which was found essential for cytokines synthesis and release [28,29].

The present study aimed at defining the role played by spinal dorsal horn microglial kinin B_1_R in a classical rat model of diabetes-induced pain neuropathy by using two inhibitors of microglial cells. Formally, were tested fluorocitrate, a specific inhibitor of microglia Krebs cycle [30], and minocycline, a broad spectrum tetracycline antibiotic, which inhibits microglia activity by preventing the translocation of the transcriptional nuclear factor kappa B (NF-κB) to its nuclear promoter [31]. The specific objectives were to: 1) determine whether microglia inhibitors can prevent thermal hyperalgesia and tactile allodynia induced by spinal activation of B_1_R with the selective agonist des-Arg^9^-BK in STZ-diabetic rats; 2) compare the acute inhibition of B_1_R and microglial function on tactile and cold allodynia; 3) determine the effect of microglia inhibition on the expression of B_1_R and pro-inflammatory markers (IL-1β, TNF-α, TRPV1) by real-time RT-PCR; 4) correlate changes of B_1_R mRNA levels with those of B_1_R binding sites by quantitative autoradiography; 5) measure the immunoreactivity of Iba-1 as marker of microglia. This study was carried out in the early phase of diabetes (4 days post-STZ) when allodynia, spinal kinin B_1_R up-regulation and thermal hyperalgesia upon intrathecal injection of des-Arg^9^-BK were found to occur [7,23].

## Methods

### Animals and treatments

All research procedures and the care of the animals were in compliance with the guiding principles for animal experimentation as enunciated by the Canadian Council on Animal Care and were approved by the Animal Care Committee of our University. Male Sprague-Dawley rats (225-250 g, Charles River, St-Constant, Que, Canada) were housed two per cage, under controlled conditions of temperature (23°C) and humidity (50%), on a 12 h light-dark cycle and allowed free access to normal chow diet (Charles River Rodent) and tap water.

### Intrathecal implantation of catheter

Four days after arrival, rats were chronically implanted, under isoflurane anaesthesia, with an indwelling intrathecal (i.t.) polyethylene catheter (PE-10; Intramedic, Clay Adams, NJ, USA) at the vertebral mid-lumbar level (L2 to L4) through an incision made in the dura at the atlanto-occipital junction [7]. This catheter was used for intrathecal drug injections. On the day of surgery and for the 2 subsequent days, rats received antibiotics trimethoprim and sulphadiazine (Tribrissen 24%, 30 mg/kg, s.c., Schering Canada Inc., Pointe Claire, Que, Canada) and the analgesic ketoprofen (Anafen, 5 mg/kg, s.c., Merial Canada Inc., Baie d'Urfé, Que, Canada).

### STZ treatment

Five days after intrathecal implantation, rats were injected under a low light condition with freshly prepared STZ (65 mg/kg, i.p.; Zanosar, McKesson, Montreal, Que, Canada). Age-matched controls were injected with vehicle (sterile saline 0.9%, pH. 7.0). Blood glucose was measured with a commercial blood glucose-monitoring kit (Accusoft; Roche Diagnostics, Laval, Que, Canada) from a drop of blood obtained from the tail vein, in non-fasting animals. Only STZ-treated rats whose blood glucose concentration was higher than 20 mM at day 4 were used and considered as diabetic.

### Tactile allodynia

Four days after STZ treatment, tactile allodynia was assessed by measuring the hindpaw withdrawal threshold to the application of a calibrated series of 6 von Frey filaments (bending forces of 2, 4, 6, 8, 10 and 15 g) (Stoelting, Wood Dale, IL, USA) using a modification of the up-down method [32]. Starting with the filament that has the lowest force (2 g), the filament was applied perpendicularly to the mid-plantar surface with sufficient force to cause the filament to buckle slightly. Brisk withdrawal or paw flinching was considered as the positive response. Each filament was applied five times to each paw (for 6-8 s per stimulation, with an inter stimulus interval of 1-2 min). Minimum recording of 5 positive responses (50%) out of 10 stimulations for both paws was considered to be the threshold (in grams). Absence of a response (less than 5 withdrawals) prompted use of the next graded filament of increasing weight. The cut-off of a 15 g filament was selected as the upper limit for testing, since stiffer filaments tended to raise the entire limb rather than to buckle, substantially changing the nature of the stimulus. Control rat withdrawal threshold was between 10 and 12 g.

### Cold allodynia

Four days after STZ treatment, cold allodynia was assessed using the acetone drop method [33]. An acetone bubble formed at the end of a standard plastic syringe was placed to the plantar surface of the hindpaws. Acetone was applied five times to each paw at intervals of 3-5 min. Control rats either ignored the stimulus or occasionally responded with a small and brief withdrawal. Allodynic rats responded with prompt and intense paw withdrawal or escape behaviour to acetone application. The frequency of paw withdrawal was expressed as a percentage (the number of paw withdrawals ÷ number of trials × 100).

### Tail-flick test

Intrathecally implanted rats were housed permanently in the testing laboratory under continuous light to prevent the endogenous release of opoids which could alter the nociceptive threshold [34]. Testing began four days after STZ treatment by placing awake rats in a plastic restraining box. The nociceptive threshold was taken as the reaction time to remove the tail from above a source of noxious radiant heat. The intensity of the heat stimulus was set to elicit a tail-flick within 8-10 s. A 25 s cut-off time was used to prevent tissue damage. Four groups of rats (control, STZ-vehicle, STZ-fluorocitrate and STZ-minocycline, n = 5) were tested for a maximum of three consecutive days and euthanized with CO_2 _inhalation after the last test. Fluorocitrate (1 nmol, i.t.) or minocycline (10 mg/kg, i.p.) was administered 3 h prior to the tail- flick test protocol. During the first two days, rats received only 20 μl of artificial cerebrospinal fluid (aCSF) as training experiments and to ensure that the intrathecal catheter is patent. On the following day, each testing trial lasted 1 h and consisted of 13 measurements of tail-flick latency, spaced by 5 min intervals. The initial three measurements were used to determine baseline latency. One minute prior to the 4th reading (t = 15 min) the vehicle (aCSF) was intrathecally injected. Fifteen min later, the tested B_1_R agonist (des-Arg^9^-BK, 9.6 nmol or 10 μg) was injected through the same route (t = 30 min) and its effect on the tail-flick latency was measured 1 min later and on three subsequent readings. Then substance P (6.6 nmol or 10 μg) was injected intrathecally to assess the specificity of B_1_R blockade on microglia.

### Spinal cord preparation

For autoradiography, 4-day STZ-diabetic rats (without intrathecal catheter) were anesthetised with CO_2 _inhalation and then decapitated. Thoracic spinal cord (T3-T7) was removed and frozen in 2-methylbutane (cooled at -40°C following exposure to liquid nitrogen) and stored at -80°C. For microscopy, rats were anesthetized with sodium pentobarbital (80 mg/kg, i.p.) and perfused transcardially with 0.1 M PBS (pH 7.4) (300 ml in 3 min), followed by 4% paraformaldehyde in PBS (500 ml in 5 min). The thoracic spinal cord (T3-T7) was removed and post-fixed by immersion in paraformaldehyde (60 min at 4°C) and washed in PBS [35]. Few days later, spinal cord segments were mounted in a gelatin block and serially cut into 20-μm thick coronal sections with a cryostat. Sections were then thaw-mounted on Fisherbrand Superfrost disposable microscope slides (Fisher Scientific, ON, Canada) and kept at -80°C for 1 month to allow the adhesion of sections to the coverslip glasses.

### Quantitative autoradiography

The radioligand for kinin B_1_R, HPP-desArg^10^-Hoe140 (3-(4 hydroxyphenyl) propionyl-desArg^9^-D-Arg^0^(Hyp^3^,Thi^5^,D-Tic^7^,Oic^8^)Bradykinin) was synthesized at the National Research Council of Canada (NRC, Montreal, Que, Canada) and iodinated by the chloramine T method [36]. Quantitative *in vitro *autoradiography was conducted as described previously [23]. Briefly, sections were incubated at room temperature for 90 min in 25 mM PIPES-NH_4_OH buffer (pH 7.4) containing: 1 mM 1,10-phenanthroline, 1 mM dithiothreitol, 0.014% bacitracin, 0.1 mM captopril, 0.2% bovine serum albumin (protease free) and 7.5 mM magnesium chloride in the presence of 200 pM of (^125^I)-HPP-desArg^10^-Hoe 140 (specific activity: 2000 cpm/fmol or 1212 Ci/mmol). Non-specific binding was determined in the presence of 1 μM of unlabeled B_1_R antagonist: R-715 (AcLys(D-βNal^7^,Ile^8^)des-Arg^9^-BK) [8]. At the end of the incubation period, slides were transferred sequentially through four rinses of 4 min each in 25 mM PIPES (pH 7.4; 4°C) dipped for 15 s in distilled water (4°C) to remove the excess of salts, and then air-dried. Kodak Scientific Imaging Films BIOMAX ™ MR^® ^(Amersham Pharmacia Biotech Canada) were juxtaposed onto the slides in the presence of (^125^I)-microscales and exposed at room temperature for 6 days. The films were developed (GBX developer) and fixed (GBX fixer). Autoradiograms were quantified by densitometry using an MCID™ image analysis system (Imaging Research, St. Catharines, ON, Canada). A standard curve from (^125^I)-microscales were used to convert density levels into fentomoles per milligram of proteins [37]. Specific binding was determined by subtracting values of nonspecific binding from that of total binding.

### Confocal microscopy

On the day of experiments, slides of spinal cords were briefly (10 min) thawed at room temperature (RT) to enhance sections adhesion. Then, sections were washed for 10 min in 0.1 M PBS buffer (pH 7.4) and incubated for 30 min (RT) in blocking buffer (PBS containing 3% bovine serum albumin, 3% donkey serum and 0.5% triton X-100) to prevent non-specific labeling. Sections were incubated 2 h (RT) with the blocking buffer containing 2 μg/ml of the primary antibody rabbit anti-Ionized calcium binding adapter molecule-1 (anti-Iba-1, Wako, Richmond, VA, USA) [22]. Sections were washed 3 times (5 min) and incubated 90 min (RT) with PBS containing 1:500 of the secondary antibody rhodamine anti-rabbit (ext: 550 nm; em: 570 nm) (Chemicon, Hornby, ON, Canada). Slides were washed 3 times (5 min), mounted with coverslip, fixed with Vectashield (Vector Laboratories, Burlington, ON, Canada) 12 h (RT) and stored at -4°C for up to 1 month. The examination was limited to the dorsal horn under confocal microscope (Leica Confocal microscope, Richmond Hill, ON, Canada), using the krypton laser (568 nm).

### Quantification of Iba-1 immunolabelling

Leica confocal program was used to quantify the mean pixel energy of Iba-1 in the spinal dorsal horn. A minimum of 4 slides from 4 different rats were measured. In everyone slide, relative background energy was subtracted from the mean pixel energy.

### SYBR green-based quantitative RT-PCR

Four days after injection of STZ, rats were anesthetised with CO_2 _inhalation and then decapitated. The upper thoracic spinal cord (T1-T2) was isolated and approximately 10 mg of tissue were put in RNA*later *stabilization reagent (QIAGEN, Valencia, CA, USA). Total RNA was extracted from tissue according to the manufacturer's instructions. First-strand cDNA synthesized from 400 ng total RNA with random hexamer primers was used as the template for each reaction with the QuantiTect Rev Transcription Kit (QIAGEN). SYBR Green-based real-time quantitative PCR using Mx3000 p device for signal detection (Stratagene, La Jolla, CA, USA) was performed as described [22]. PCR was performed in SYBR Green Master mix (QIAGEN) with 300 nM of each primer. For standardization and quantification, rat 18S was amplified simultaneously. The primer pairs were designed by Vector NTI software (Table 1). PCR conditions were as follows: 95°C for 15 min, followed by 46 cycles at 94°C for 15 s, 60°C for 30 s and 72°C for 30 s. The cycle threshold (Ct) value represents the cycle number at which a fluorescent signal rises statistically above background. The relative quantification of gene expression was analyzed by the 2^-ΔΔCt ^method [38].

**Table 1:** PCR primer pairs used in this study

	Sequences			Position			Gen Bank
18S forward	5'	TCA ACT TTC GAT GGT AGT CGC CGT	3'	363	-	386	X01117
18S reverse	5'	TCC TTG GAT GTG GTA GCC GTT TCT	3'	470	-	447	

B_1 _receptor forward	5'	GCA GCG CTT AAC CAT AGC GGA AAT	3'	367	-	391	NM_030851
B_1 _receptor reverse	5'	CCA GTT GAA ACG GTT CCC GAT GTT	3'	478	-	454	

IL-1β forward	5'	TGT CAC TCA TTG TGG CTG TGG AGA	3'	247	-	270	NM_031512
IL-1β reverse	5'	TGG GAA CAT CAC ACA CTA GCA GGT	3'	411	-	388	

TNF-α forward	5'	ATG ATC CGA GAT GTG GAA CTG GCA	3'	160	-	183	NM_012675
TNF-α reverse	5'	AAT GAG AAG AGG CTG AGG CAC AGA	3'	257	-	234	

TRPV1 forward	5'	GCA CAA TGG GCA GAA TGA CAC CAT	3'	575	-	598	NM_031982
TRPV1 reverse	5'	GGC ATT GAC AAA CTG CTT CAG GCT	3'	656	-	633	

### Drugs

The kinin B_1_R was blocked with the orally active, non-peptide B_1_R antagonist, SSR240612, which crosses readily the blood-brain barrier [39]. It was kindly provided by Sanofi-Aventis R&D (Montpellier, France). R-715 was also used as B_1_R antagonist as it does not cross the blood-brain barrier due to its peptide nature [8]. It was generously obtained from Dr Fernand Gobeil Jr. (Pharmacology, Sherbrooke University, Sherbrooke, Que, Canada). SSR240612 (10 mg/kg) was administered by gavage (volume of 1 ml by 100 g of rat body weight) while R-715 (10 mg/kg) was given intraperitoneally. Both antagonists at this dose blocked allodynia in a rat model of type 2 diabetes [20,21]. SSR240612 and R-715 were also administered intrathecally at a single dose of 10 μg to better dissociate the spinal and peripheral contribution of B_1_R in the functional tests. Microglial cells were blocked with fluorocitrate (1 nmol, i.t.), a selective inhibitor of the Krebs cycle enzyme aconitase [40,41] and with minocycline (10 mg/kg, i.p.), an antibiotic preventing the translocation of microglia NF-κB to its nuclear promoter [31]. The B_1_R agonist, des-Arg^9^-BK (9.6 nmol, i.t.) caused hyperalgesia in the tail-flick test in STZ rats [7] while the NK-1 receptor agonist, substance P (SP) (6.6 nmol, i.t.) caused hyperalgesia in the tail-flick test in control rats [42]. Both agonists were purchased from Bachem Bioscience Inc. (King of Prussia, PA, USA). For intraperitoneal injections, minocycline and R-715 were dissolved in 0.9% sterile saline. For intrathecal injections, des-Arg^9^-BK, SP, R-715 and fluorocitrate were dissolved in aCSF. For all treatments, SSR240612 was dissolved in dimethylsulphoxide (0.5%), and then ethanol (5%) and Tween-80 (5%) were added in this sequence [20]. The solution was completed in distilled water. Fluorocitrate, minocycline and all other reagents were purchased from Sigma-Aldrich Canada, Ltd (Oakville, ON, Canada).

### Statistical analysis

All data were expressed as the means ± S.E.M. obtained from *n *rats. In the tail-flick test, data were calculated as a percentage of the maximum possible effect (% MPE) according to the following formula: % MPE = 100 × (drug latency minus baseline latency) ÷ (cut-off time minus baseline latency) [7]. The baseline latency corresponds to the average of the first three measurements. Statistical significance was determined with Student's *t*-test for paired samples or one-way analysis of variance (ANOVA) followed by post-hoc Bonferonni test for multiple comparisons. Data for allodynia were analysed with the non-parametric Kruskal-Wallis post-test. Only probability (P) values less than 0.05 were considered to be statistically significant.

## Results

### Influence of microglia inhibitors and B_1_R antagonists on STZ-induced hyperglycemia

**Figure 1:**
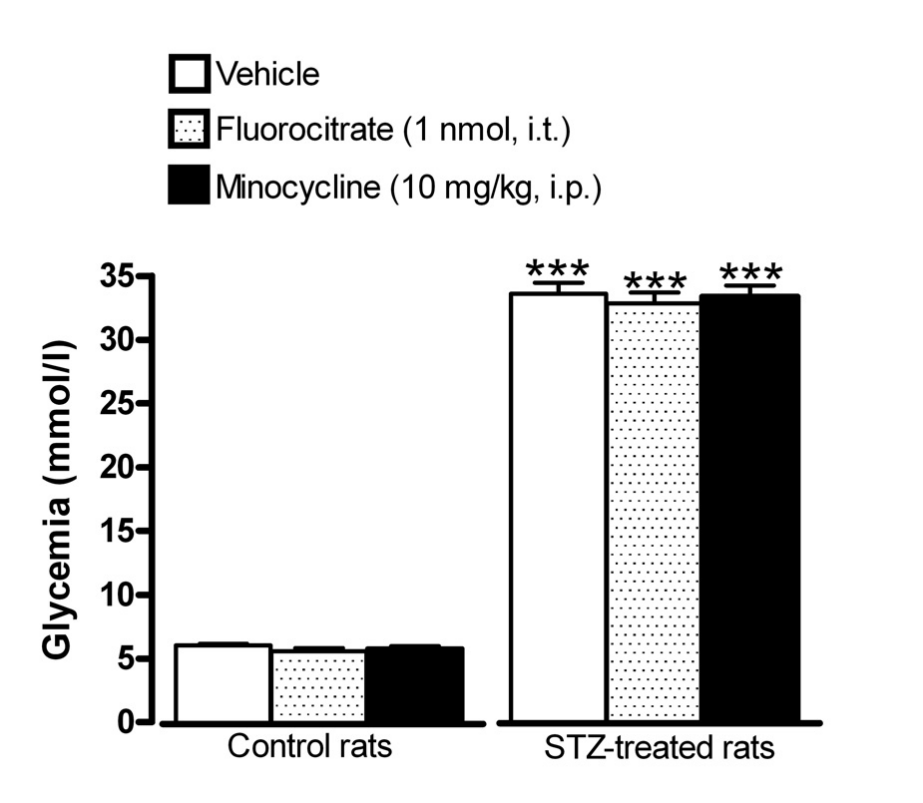
**Effect of microglia inhibitors administered 3 h earlier on blood glucose concentration in control and 4-day STZ-diabetic rats**. Data are the mean ± S.E.M. of 5 rats in each group. Statistical comparison to control rats is indicated by ***P < 0.001.

**Figure 2:**
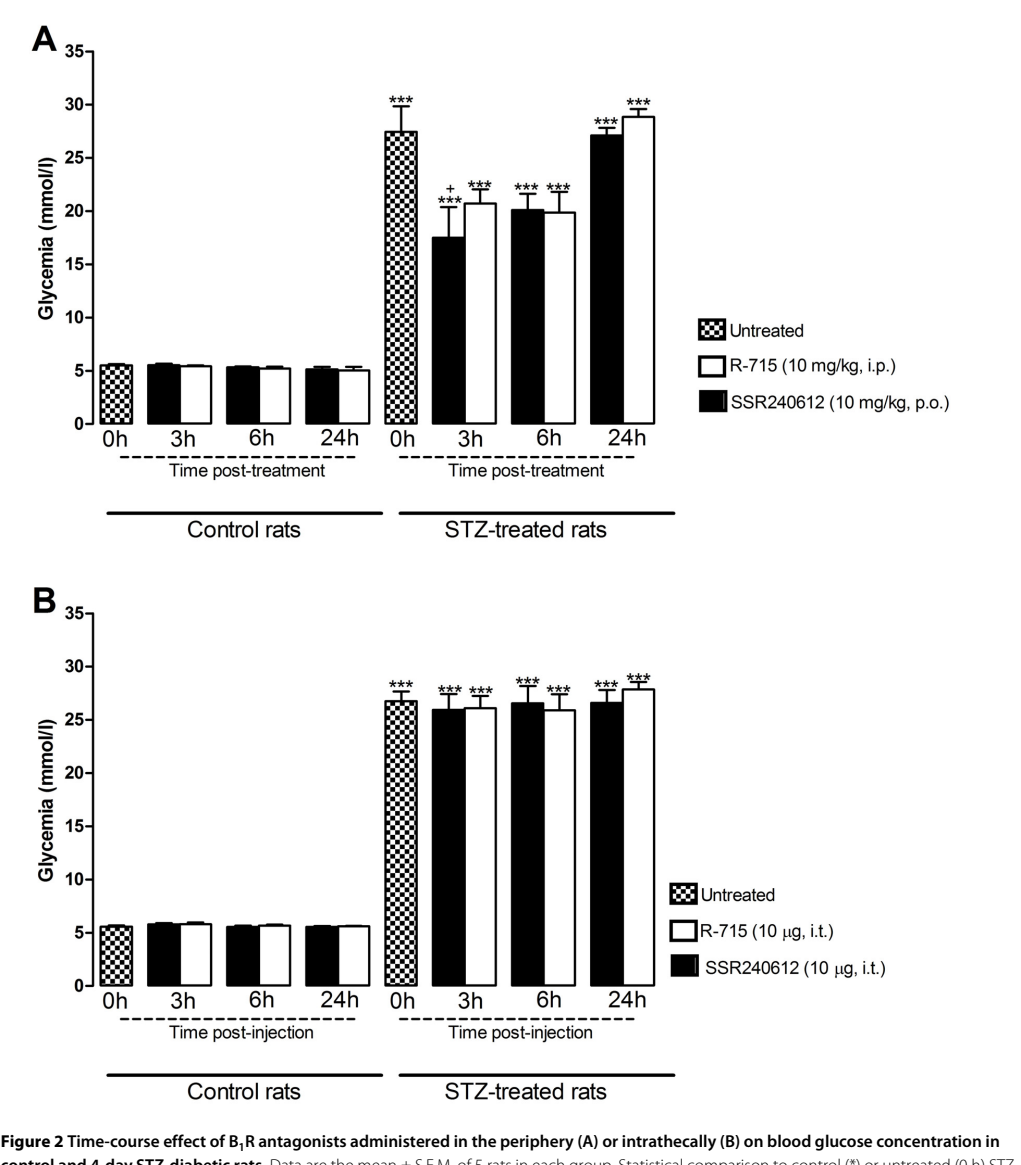
**Time-course effect of B_1_R antagonists administered in the periphery (A) or intrathecally (B) on blood glucose concentration in control and 4-day STZ-diabetic rats. **Data are the mean ± S.E.M. of 5 rats in each group. Statistical comparison to control (*) or untreated (0 h) STZ-treated rats (+) is indicated by ^+ ^P < 0.05, ***P < 0.001.

As depicted in Figure 1, rats which received STZ (65 mg/kg, i.p.) 4 days earlier displayed a significant increase of blood glucose concentration compared with vehicle-matched control rats. Blood glucose levels in control and STZ-treated rats were not affected by fluorocitrate (1 nmol, i.t.) or minocycline (10 mg/kg, i.p.) injected 3 h earlier (Figure 1). SSR240612 (10 mg/kg, p.o.) reduced significantly hyperglycemia in STZ-treated rats at 3 h post-gavage; the inhibitory effect of the B_1_R antagonist was not significant at 6 h and was completely resolved at 24 h. A similar pattern of anti-hyperglycemia was seen with R-715 (10 mg/kg, i.p.), yet the inhibitory effect did not reach statistical significance (Figure 2-A). Either antagonist did not affect glycemia in control rats. Intrathecally administered R-715 and SSR240612 (10 μg) failed to alter blood glucose levels in both control and STZ-diabetic rats (Figure 2-B).

### Effect of microglia inhibitors on Iba-1 microglial immunoreactivity

**Figure 3:**
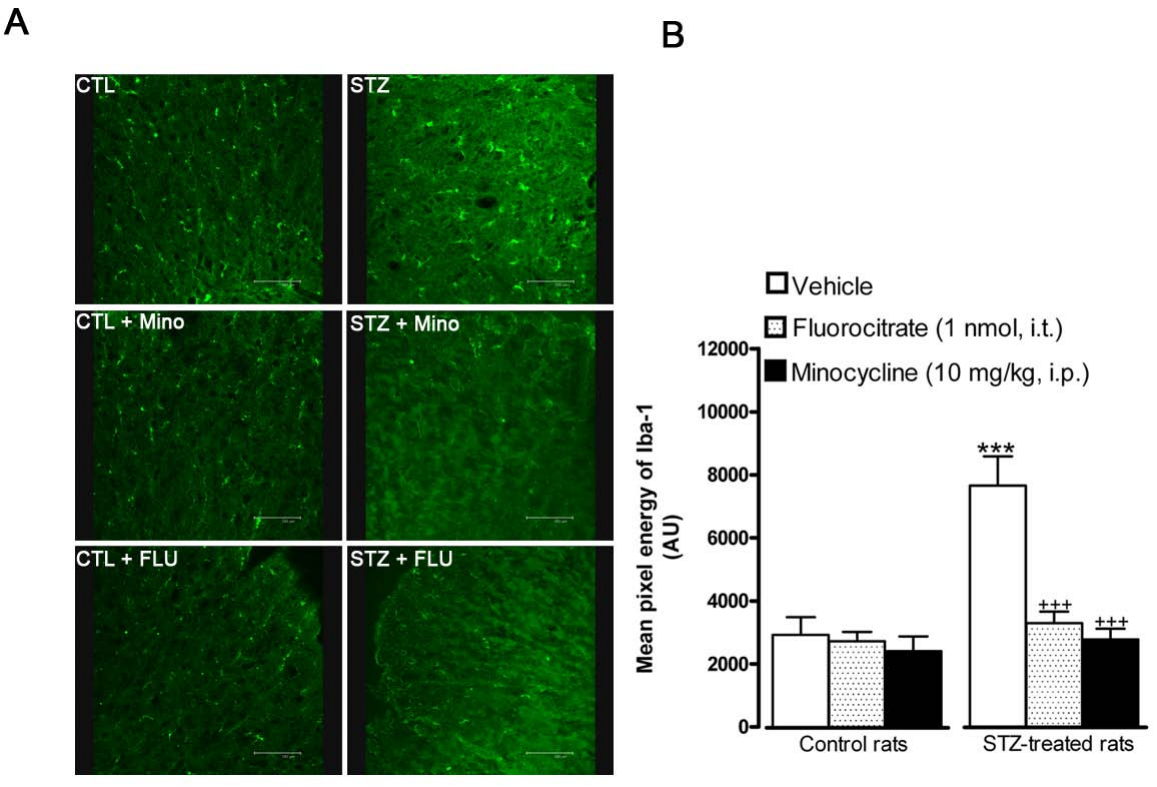
**Effect of microglia inhibitors administered 3 h earlier on Iba-1 microglial immunoreactivity in the spinal dorsal horn of control and 4-day STZ-diabetic rats**. Shown are (A) confocal microscopy pictures of microglial cells, and (B) the quantification of the mean pixel energy of Iba-1 (in arbitrary unit, AU). Scale bar = 100 μm. Data are the mean ± S.E.M of 4 pictures per rat from 4 rats in each group. Background mean energy was subtracted for each picture. Statistical comparison to control (*) or STZ rats treated with vehicle (^+^) is indicated by *** ^+++ ^P < 0.001.

A quantitative immunolabelling with a specific immunomarker of microglia Iba-1 was employed to validate the use of minocycline and fluorocitrate as inhibitors of microglia activity. As shown in Figure 3-A, immunoreactivity to Iba-1 was much more striking in the spinal dorsal horn of STZ-diabetic rats than in matched control spinal dorsal horn. Immunoreactive microglial cells in STZ spinal cord were more numerous, thicker and displayed higher mean pixel energy than microglia of control spinal dorsal horn. Importantly, treatment with fluorocitrate or minocycline reversed and normalized the enhanced Iba-1 immunoreactivity in STZ spinal cord microglia (Figure 3-B). The same treatment with fluorocitrate or minocycline had no effect on the mean pixel energy of Iba-1 in control spinal dorsal horn.

### Effect of microglia inhibitors on mRNA levels of B_1_R, TRPV1, IL-1β and TNF-α in STZ thoracic spinal cord

Since pro-inflammatory cytokines are involved in the induction of B_1_R and that TRPV1 is associated with pain neuropathy, this series of experiments was carried out to measure their expression in STZ-diabetic rats. As illustrated in Table 2, mRNA levels of these markers were markedly increased (IL-1β > B_1_R > TNF-α > TRPV1) in the spinal cord of STZ-diabetic rats. Treatment with fluorocitrate (1 nmol, i.t.) or minocycline (10 mg/kg, i.p.), 3 h earlier, markedly reduced the enhanced mRNA to values not significantly different from control levels.

**Table 2:** Gene expression of various markers in thoracic spinal cord of STZ-diabetic rats

	gene mRNA/18S mRNA (fold change)			
	B_1_R	TRPV1	IL-1β	TNF-α
Control	1.0 ± 0.4	1.0 ± 0.1	1.0 ± 0.6	1.0 ± 0.6
				
STZ	27.3 ± 8.7 *	3.1 ± 0.4 *	77.9 ± 19.6 *	6.0 ± 2.1 **
				
STZ + Fluorocitrate	2.7 ± 1.1	2.3 ± 0.3 +	10.1 ± 7.9	0.9 ± 0.4 +
				
STZ + Minocycline	3.0 ± 1.3	2.6 ± 0.2 +	8.9 ± 4.2	2.1 ± 0.7 +

Effect of a 3 h pre-treatment with fluorocitrate (1 nmol, i.t.) or minocycline (10 mg/kg, i.p.) on gene expression of inflammatory markers in thoracic spinal cord of STZ-diabetic rats. Data represent the mean ± S.E.M. of 5-6 rats in each group. Statistical comparison to control (*) and STZ-diabetic rats without microglia inhibitors (+) is indicated by * ^+ ^P < 0.05; **P < 0.01.

### Effect of fluorocitrate on B_1_R specific binding sites in STZ thoracic spinal cord

**Figure 4:**
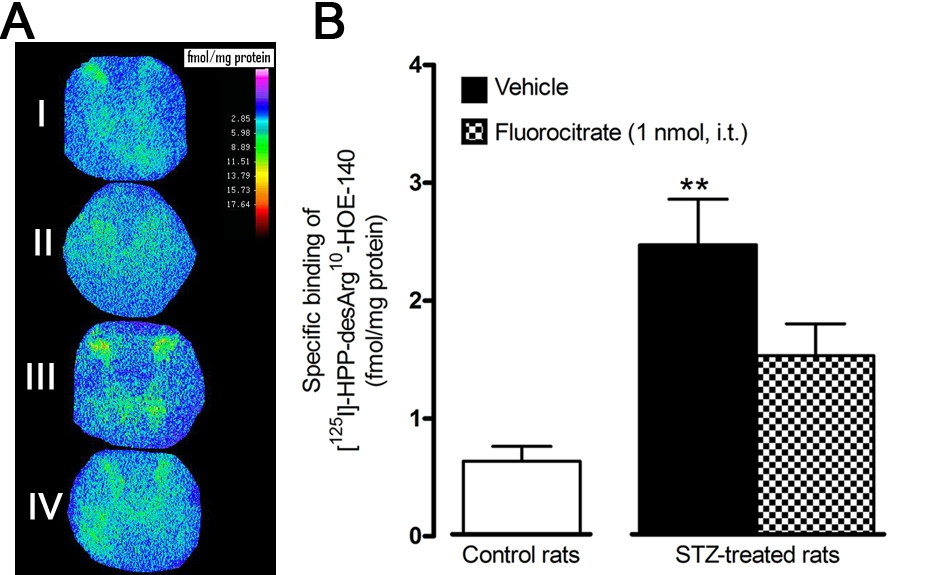
**Effect of microglia inhibitors administered 3 h earlier on B_1_R binding sites in the thoracic spinal cord of control and 4-day STZ-diabetic rats**. Shown in (A) are autoradiograms of Control (I), Non-specific (II), STZ (III) and STZ + fluorocitrate (IV), and in (B) are the quantification of specific densities of B_1_R binding sites in spinal dorsal horn (Laminae I-III). Data are the mean ± S.E.M. of 5 to 6 rats in each group. Statistical comparison to control rats is indicated by **P < 0.01.

A low density of specific kinin B_1_R binding sites was detected in the spinal cord dorsal horn (laminae I-III) of control rats (0.637 fmol/mg protein). This value was increased by 4-fold in spinal cord of STZ-treated rats (2.474 fmol/mg protein). Fluorocitrate (1 nmol, i.t.) administered in STZ-diabetic rats, 3 h prior sacrifice, reduced (-38%) the increased density of specific B_1_R binding sites induced by diabetes to values (1.532 fmol/mg protein) not significantly different from control density (Figure 4).

### Effect of microglia inhibitors on thermal hyperalgesia mediated by des-Arg^9^-BK

**Figure 5:**
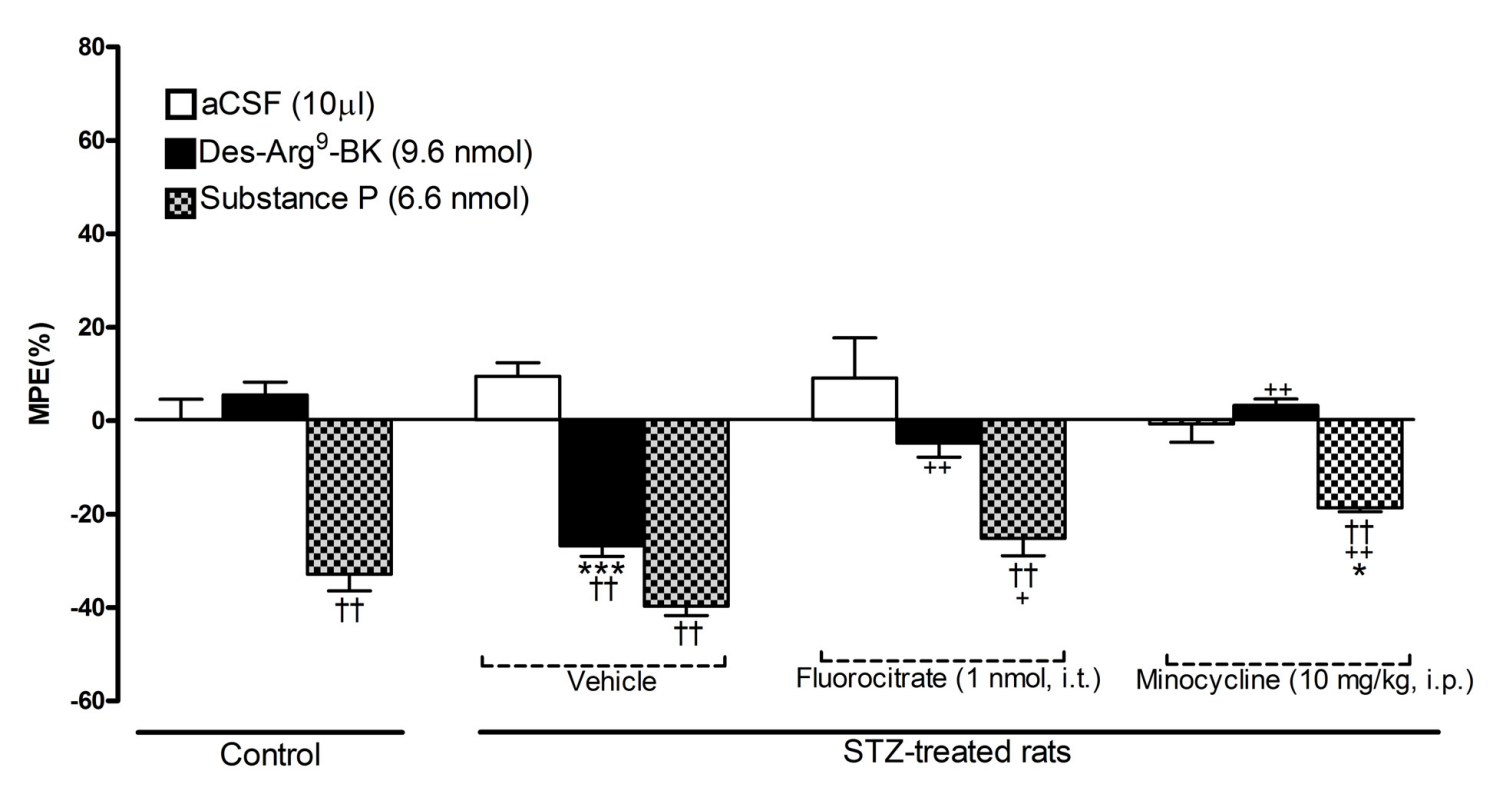
**Effect of microglia inhibitors administered 3 h earlier on tail-flick reaction time (MPE %) in control and 4-day STZ-diabetic rats**. Shown are the maximal responses measured 1 min after intrathecal injection of either aCSF, 9.6 nmol des-Arg^9^-BK or 6.6 nmol SP. Data are the mean ± S.E.M. of 5 rats in each group. Within groups, statistical comparison to aCSF is indicated by ^††^P < 0.01, while statistical comparison to the same agonist in the control group (*) or in STZ + vehicle (+) is indicated by * ^+ ^P < 0.05; ^++ ††^P < 0.01; ***P < 0.001.

As shown in Figure 5, control rats treated with SP (6.6 nmol, i.t.) showed a significant decrease in reaction time to thermal stimulation at 1 min post-injection (-35%) in comparison with aCSF. This response was not significantly increased (-40%) in STZ-diabetic rats. Whereas des-Arg^9^-BK (9.6 nmol, i.t.) had no significant effect in control rats, a significant decrease in reaction time (-30%) occurred at 1 min post-injection in STZ-diabetic rats. A 3 h pre-treatment with fluorocitrate (1 nmol, i.t.) or minocycline (10 mg/kg, i.p.) abolished the hyperalgesic response to des-Arg^9^-BK (-5% and + 2%, respectively). Both fluorocitrate and minocycline reduced significantly SP-induced hyperalgesia in STZ-diabetic rats. This result indicates that microglia inhibition may partly contribute to SP-induced hyperalgesia in STZ-diabetic rats. Baseline values of the thermal nociceptive threshold in control rats (9.04 ± 0.79; n = 5) were not affected (P > 0.05) at this early stage of STZ-induced diabetes (9.16 ± 0.45; n = 5) neither by fluorocitrate (9.07 ± 0.93; n = 5) nor minocycline (9.12 ± 0.73; n = 5) treatment.

### Effect of microglia inhibitors and B_1_R antagonists on allodynia induced by STZ

**Figure 6:**
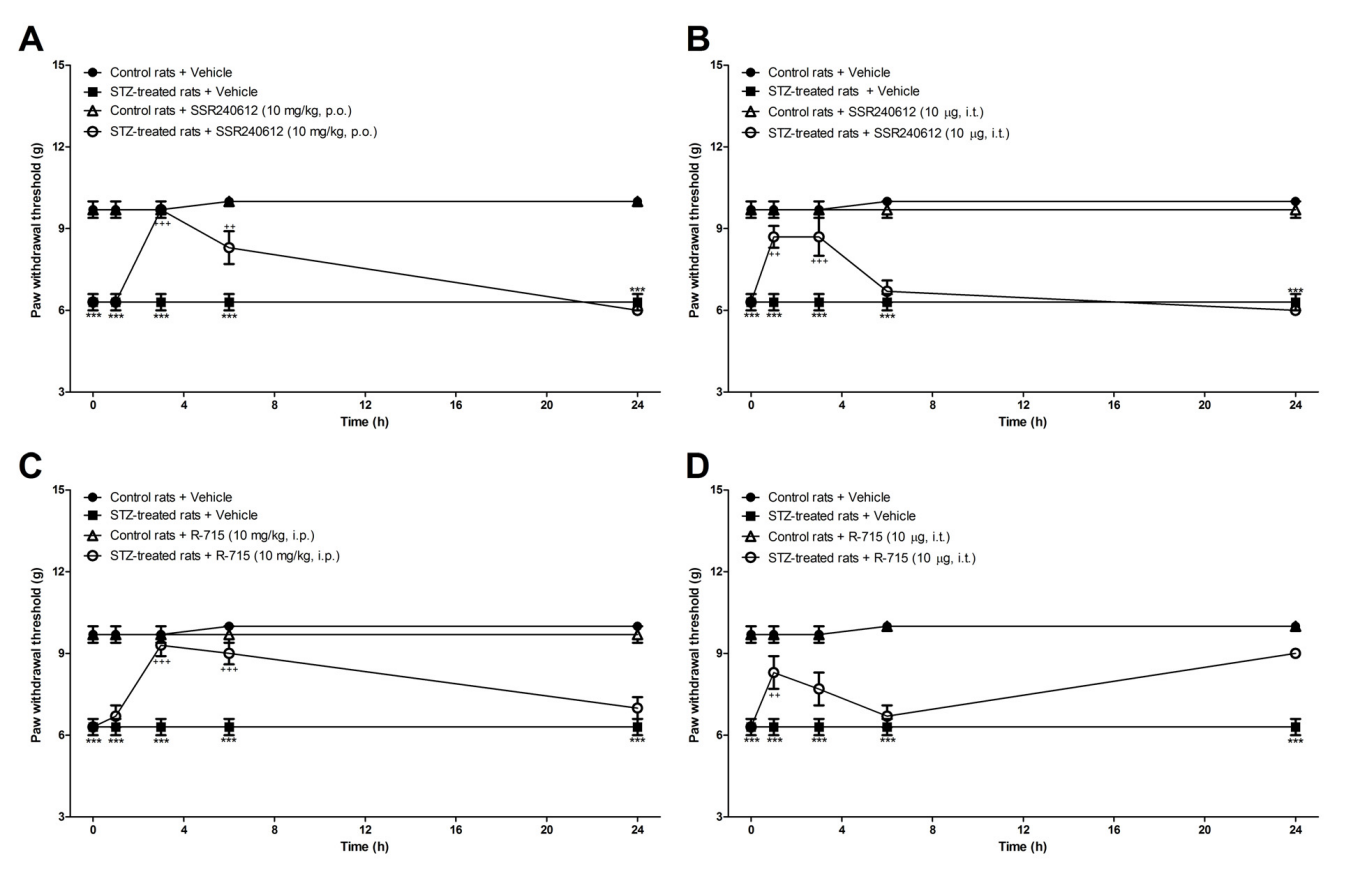
**Time-course of the inhibitory effect of B_1_R antagonists administered in the periphery (A, C) or intrathecally (B, D) on spontaneous paw withdrawal threshold to tactile stimulation (g) in control and 4-day STZ-diabetic rats**. All treatments were given at time 0 h. Data are the mean ± S.E.M. of 4 to 12 rats in each group. Statistical comparison to control + vehicle (*) and STZ + vehicle (^+^) is indicated by ^++^P < 0.01; *** ^+++^P < 0.001.

**Figure 7:**
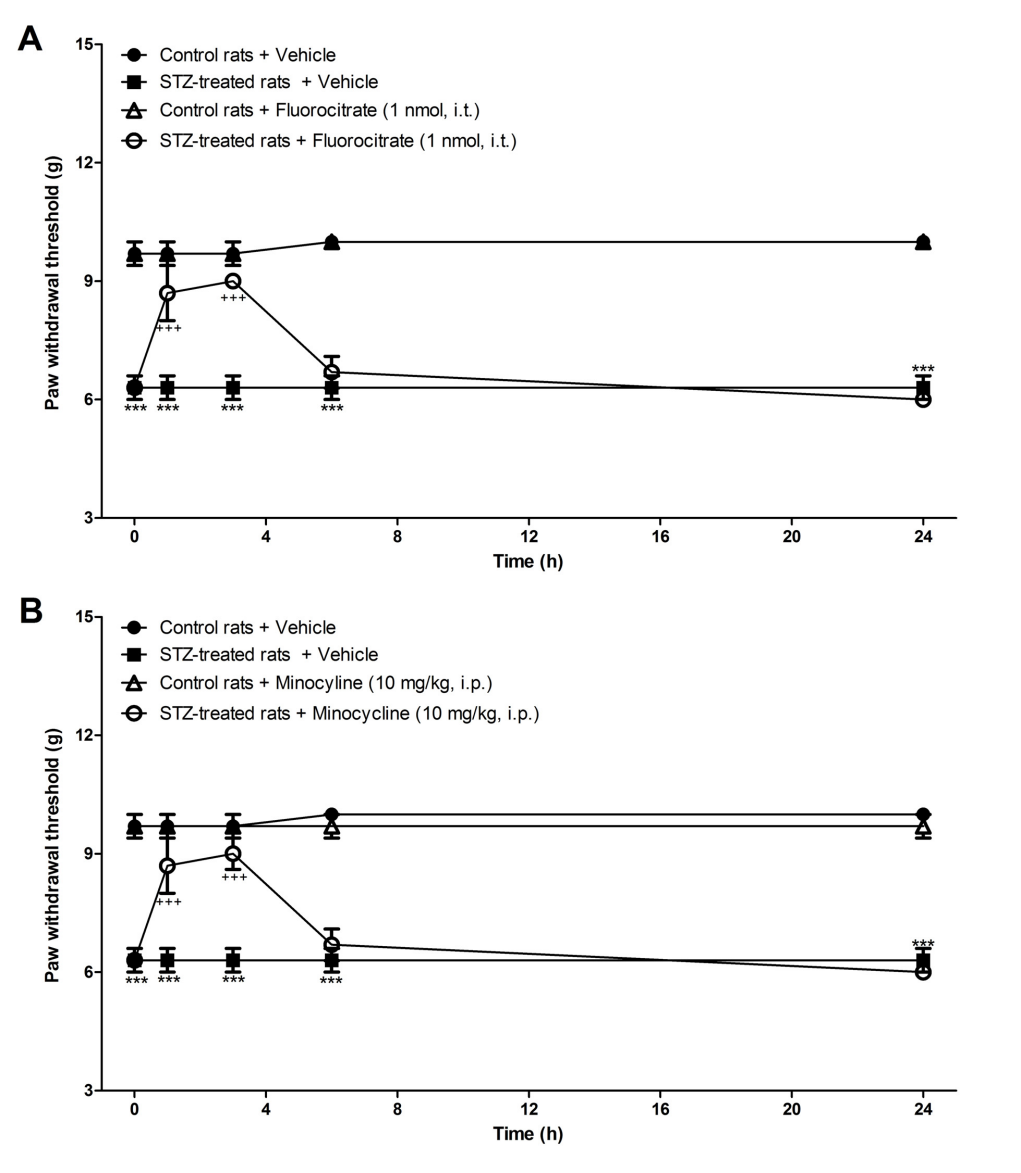
**Time-course of the inhibitory effect of fluorocitrate (A) and minocycline (B) on spontaneous paw withdrawal threshold to tactile stimulation (g) in control and 4-day STZ-diabetic rats**. All treatments were given at time 0 h. Data are the mean ± S.E.M. of 4 rats in each group. Statistical comparison to control + vehicle (*) and STZ + vehicle (^+^) is indicated by *** ^+++^P < 0.001.

**Figure 8:**
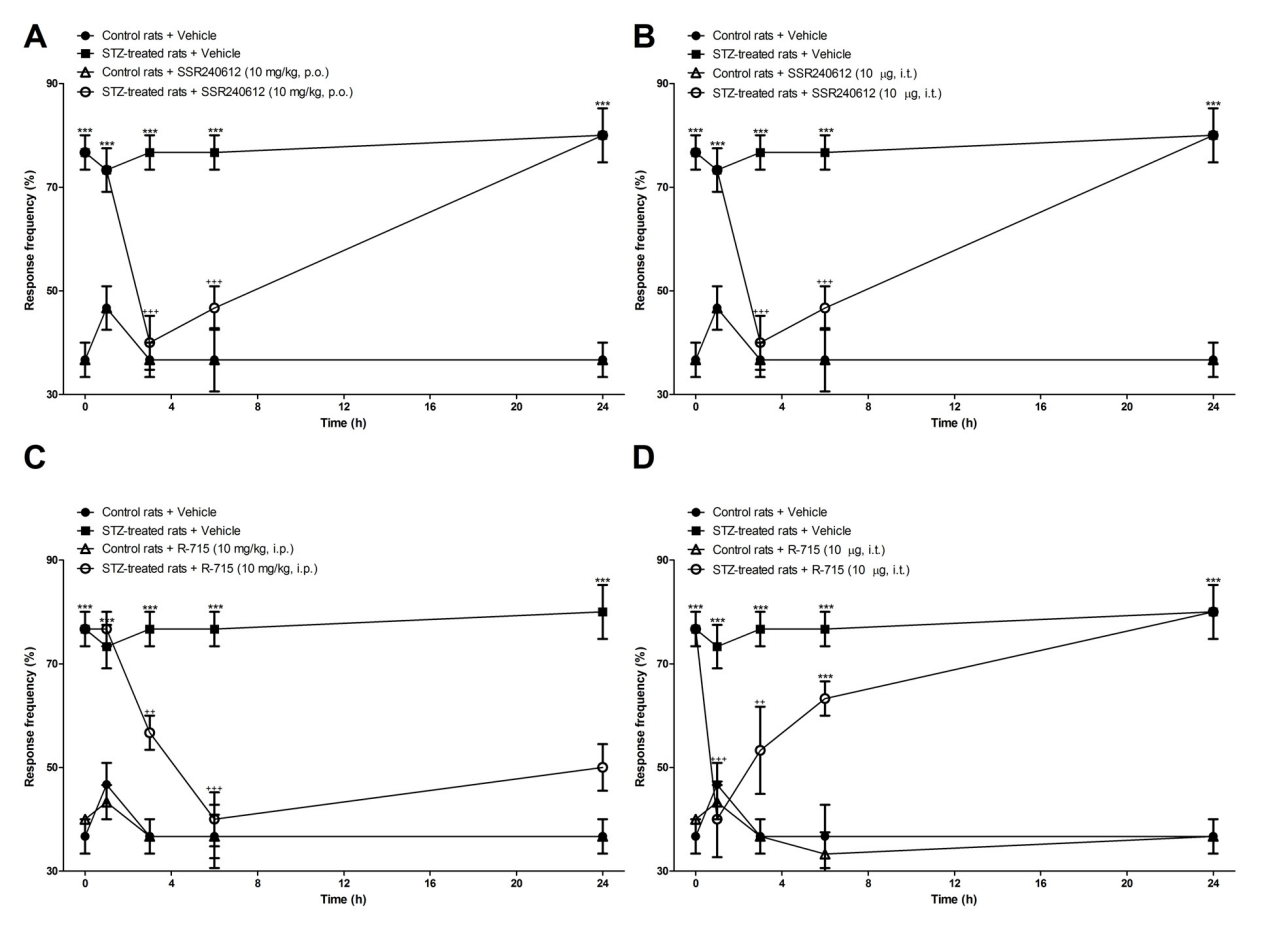
**Time-course of the inhibitory effect of B_1_R antagonists administered in the periphery (A, C) or intrathecally (B, D) on paw withdrawal response frequency (%) to cold stimulation in control and 4-day STZ-diabetic rats**. All treatments were given at time 0 h. Data represent the mean ± S.E.M. of 4 to 12 rats in each group. Statistical comparison to control + vehicle (*) and STZ + vehicle (^+^) is indicated by ^++^P < 0.01; *** ^+++^P < 0.001.

**Figure 9:**
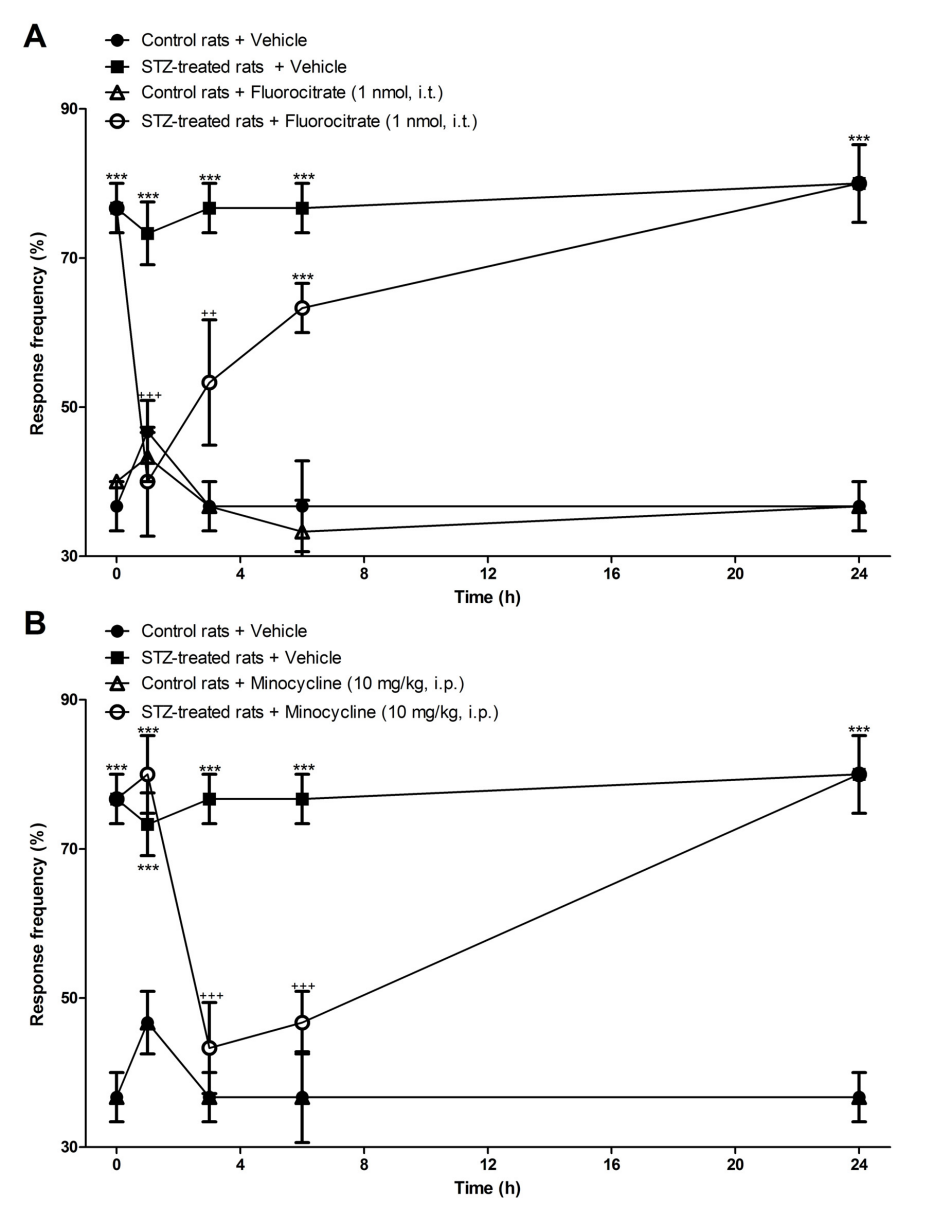
**Time-course of the inhibitory effect of fluorocitrate (A) and minocycline (B) on paw withdrawal response frequency (%) to cold stimulation in control and 4-day STZ-diabetic rats**. All treatments were given at time 0 h. Data are the mean ± S.E.M. of 4 rats in each group. Statistical comparison to control + vehicle (*) and STZ + vehicle (^+^) is indicated by ^++^P < 0.01; ^+++ ^***P < 0.001.

STZ-diabetic rats presented significant tactile (Figures 6, 7) and cold allodynia (Figures 8,9) in comparison with vehicle-matched control rats. These responses were stable when the tests were performed over a period of 24 h. The B_1_R antagonists, SSR240612 and R-715, administered systemically blocked in a transient and reversible manner cold and tactile allodynia between 3 and 6 h post-treatment in STZ-diabetic rats. Intrathecal treatments with B_1_R antagonists provided more rapid and shorter inhibition of allodynia (1-3 h) than systemic treatments (3-6 h). Fluorocitrate and minocycline caused a similar pattern of inhibition on tactile allodynia at 1 and 3 h post-treatment while the inhibition of cold allodynia with microglia inhibitors lasted at least up to 6 h post-treatment. The same treatments with B_1_R antagonists or microglia inhibitors had no consequence on baseline values in control rats.

### Effect of microglia inhibitors on enhanced tactile allodynia induced by des-Arg^9^-BK in STZ-treated rats

As observed in Table 3, STZ-diabetic rats displayed significant tactile allodynia in comparison with control rats. In diabetic rats, the intrathecal stimulation of B_1_R with its native agonist (des-Arg^9^-BK, 9.6 nmol) induced a significant and transient enhancement of tactile allodynia at 10 min post-injection. This response was prevented by the inhibition of B_1_R (SSR240612, 10 μg, i.t.) and of microglia with fluorocitrate (1 nmol, i.t.) or minocycline (10 mg/kg, i.p.) injected 3 h earlier. Those treatments administered to control animals were without effect while they reversed tactile allodynia in STZ-diabetic rats.

**Table 3:** Effect of spinal cord B_1_R stimulation on tactile allodynia in STZ-diabetic rats

Tactile allodynia (g)						
	0 min	5 min	10 min	15 min	30 min	60 min

**Control rats**						
Vehicle	9.7 ± 0.3	9.7 ± 0.3	9.3 ± 0.7	9.7 ± 0.3	9.7 ± 0.3	9.7 ± 0.3
DABK (9.6 nmol, i.t.)	9.7 ± 0.3	9.7 ± 0.3	9.3 ± 0.7	9.7 ± 0.3	9.7 ± 0.3	9.7 ± 0.3
SSR240612 (10 μg, i.t.) + DABK	10.7 ± 0.3	9.7 ± 0.3	9.3 ± 0.7	9.7 ± 0.3	9.7 ± 0.3	9.7 ± 1.3
FLU (1 nmol, i.t.) + DABK	9.7 ± 0.3	9.7 ± 0.3	9.3 ± 0.7	9.7 ± 0.3	9.7 ± 0.3	9.7 ± 0.3
Mino (10 mg/kg, i.p.) + DABK	9.7 ± 0.3	9.7 ± 0.3	9.3 ± 0.7	9.7 ± 0.3	9.7 ± 0.3	9.7 ± 0.3

**Tactile allodynia (g)**						

	0 min	5 min	10 min	15 min	30 min	60 min
**STZ-treated rats**						
Vehicle	6.3 ± 0.3***	6.0 ± 0.5***	6.3 ± 0.3***	6.3 ± 0.3***	6.3 ± 0.3***	6.0 ± 0.5***
DABK (9.6 nmol, i.t.)	6.0 ± 0.5***	5.3 ± 0.4***	4.3 ± 0.3++ ***	5.3 ± 0.4***	6.0 ± 0.0***	5.7 ± 0.3***
SSR240612 (10 μg, i.t.) + DABK	9.3 ± 0.3+++	9.3 ± 0.0+++	9.3 ± 0.0+++	9.3 ± 0.3+++	9.7 ± 0.3+++	9.3 ± 0.0+++
FLU (1 nmol, i.t.) + DABK	9.7 ± 0.3+++	9.7 ± 0.3+++	9.7 ± 0.3+++	9.7 ± 0.3+++	9.3 ± 0.4+++	9.3 ± 0.4+++
Mino (10 mg/kg, i.p.) + DABK	9.7 ± 0.3+++	9.0 ± 0.4+++	9.3 ± 0.4+++	9.3 ± 0.4+++	8.7 ± 0.4+++	9.3 ± 0.4+++

Time-course effect of a single intrathecal (i.t.) injection of the B_1_R agonist, des-Arg^9^-BK (DABK, 9.6 nmol), on the paw withdrawal threshold to tactile stimulation (g) in control and 4-day STZ-diabetic rats pre-treated, 3 h earlier, with either B_1_R antagonist (SSR240612), microglia inhibitors (minocycline, fluorocitrate) or vehicle. Data represent the mean ± S.E.M. of 4 to 12 rats in each group. Statistical comparison to vehicle in control (*) and STZ-treated rats (^+^) is indicated by ^++^P < 0.01; *** ^+++^P < 0.001

## Discussion

The present findings support a primary role for spinal microglial kinin B_1_R in early pain neuropathy in a rat model of type 1 diabetes. Our study provides the first direct evidence that the B_1_R is involved in allodynia and hyperalgesia in STZ-diabetic rats through a mechanism involving the microglia. This is supported by 1- the similar pattern of inhibition of allodynia by microglia inhibitors and B_1_R antagonists, and 2- the blockade of the transient allodynic and hyperalgesic responses induced by the stimulation of spinal B_1_R (des-Arg^9^-BK) by microglia inhibitors. Because spontaneous cold and tactile allodynia in STZ-diabetic rats were reversed by intrathecal B_1_R antagonists and by peripheral administration of R-715, which does not cross the blood brain barrier, it is concluded that both spinal and peripheral B_1_R are involved in diabetic allodynia. This is consistent with the presence of B_1_R on spinal dorsal horn microglia in STZ-diabetic rats [22]. An additional argument supporting an interaction between B_1_R and microglia is the finding that fluorocitrate and minocycline reversed simultaneously the upregulation of B_1_R and pro-inflammatory cytokines (IL-1β and TNF-α) in the spinal cord. Hence, the results highlight a key role for microglia and pro-inflammatory cytokines in the induction and overexpression of B_1_R in the spinal cord.

### Validation of microglia inhibitors

Immunolabelling of Iba-1 in STZ-diabetic rats treated with minocyline and fluorocitrate confirmed the inhibition of microglia. The Iba-1 immunolabelling in the spinal dorsal horn of 4-days STZ-diabetic rats showed the characteristics of activated microglia with larger cell body, shorter and stouter processes [43,44]. In contrast, resting-state microglia with small size cytoplasm and long processes are observed in control spinal cord [43,44]. As expected, microglia inhibitors caused a marked diminution of Iba-1 immunoreativity and cell body volume. This result is in accordance with the literature showing that minocycline caused a marked reduction of Iba-1 immunoreactivity in diabetes [28]. To the best of our knowledge, this is however the first report showing a decrease of Iba-1 immunoreactivity after fluorocitrate treatment.

### B_1_R induction mechanism

We observed an upregulation of both B_1_R binding sites and mRNA in the spinal cord of STZ-diabetic rats in comparison with its low level of expression in controls. Those results are in accordance with previous studies [22,23]. Hyperglycemia associated with type 1 diabetes can activate NF-κB [45], which is known to induce B_1_R [9,10]. Moreover, oxidative stress associated with diabetes was reported to be involved in the induction of B_1_R [19,46].

We demonstrated that microglia inhibition prevented STZ-induced upregulation of B_1_R, TNF-α and IL-1β mRNA. The microglia can release pro-inflammatory mediators such as IL-1β [47], TNF-α [48] and reactive oxygen species (ROS) [49] all known to induce B_1_R through the nuclear translocation of NF-κB and the activation of its promoter [7,9,10]. Therefore, the suppression of B_1_R expression by fluorocitrate and minocycline is most likely linked to the inhibition of pro-inflammatory cytokines and ROS released from microglia. Since TRPV1 activation can also contribute to B_1_R induction [50], one cannot exclude that the inhibitory effect of microglia inhibitors on B_1_R is partly mediated by the inhibition of TRPV1 overexpression observed in STZ. However, the increased mRNA level of TRPV1 in STZ was much less striking (3-fold) than that of B_1_R (27-fold), IL-1β (78-fold) and TNF-α (6-fold). The inhibitory effect of fluorocitrate and minocycline on STZ-induced increase of TRPV1 mRNA was also quite modest.

### B_1_R spinal cord localization

A 3 h treatment with fluorocitrate normalized B_1_R mRNA level, yet specific dorsal horn B_1_R binding sites were reduced by only 38% in the spinal cord of STZ rats. This may suggest that the majority of induced and up-regulated B_1_R mRNA is associated with microglial cells. The discrepancy between mRNA and binding sites suggests that about 62% of specific B_1_R binding sites originate from outside the spinal cord such as dorsal root ganglion cells projecting to the dorsal horn. This would be consistent with the presence of B_1_R on sensory C-fibers identified with CGRP and TRPV1 specific antibodies [22]. B_1_R binding sites may also represent receptors located on bulbospinal projecting fibers and/or astrocytes. Based on this reasoning, data with fluorocitrate suggest that microglial cells bear approximately 38% of all B_1_R measured in the spinal cord of STZ-diabetic rats by autoradiography. The present study does not exclude a role for B_1_R present on sensory C-fibers and astrocytes in allodynia and thermal hyperalgesia as these spinal cord elements may interact with microglia to contribute to neuropathic pain.

### Allodynia

As early as 4 days after STZ-treatment, rats exerted significant spontaneous cold and tactile allodynia compared with control rats as already reported [28]. For the first time, we demonstrated that acute blockade of spinal cord B_1_R with SSR240612 or R-715 can reverse both types of allodynia in STZ-diabetic rats. Our results also showed that systemic blockade of B_1_R with the peptide B_1_R antagonist R-715 reversed STZ-induced allodynia, supporting the idea that peripherally expressed B_1_R may also contribute to neuropathic pain. This is in keeping with our recent results showing that tactile and cold allodynia, observed in a rat model of insulin resistance [20,21], can be prevented with either peripherally or centrally acting B_1_R antagonists.

Although it was extensively studied, the exact mechanism underlying sensory abnormalities in STZ-treated rats remained unclear [51]. Recent literature suggests that spinal dorsal horn microglia is a crucial component of STZ-induced tactile allodynia which is partly mediated by the extracellular signal-regulated protein kinase signaling [28]. Moreover, studies have implicated the activation of stress-activated mitogen-activated protein kinase (MAPK) p38 in spinal microglial cells in the rat model of plantar incision-induced mechanical allodynia [52]. Inhibition of microglia activation with Gabapentin [25] or fluorocitrate and minocycline (present study) reversed allodynia in STZ-treated rats. This is the first report showing the anti-neuropathic potential of minocyline in diabetic rats. The anti-allodynic effect of minocyline in rats was reported in the formalin test [53] and in neuropathic pain models of sciatic or spinal nerve injury [54-56]. Likewise, fluorocitrate could reverse allodynia in various models of polyneuropathy, including that induced by sciatic nerve injury [41], intrathecal ATP [40] and intrathecal HIV-1 envelope glycoprotein gp120 [57]. Thus, this is also the first demonstration that fluorocitrate can reverse diabetic allodynia.

### Allodynia induced by spinal cord B_1_R stimulation

Importantly, this study presented the first evidence that spinal cord activation of B_1_R with its native agonist enhanced tactile allodynia in STZ-diabetic rats. This effect was prevented by a pre-treatment with an antagonist of B_1_R or by microglia inhibition, providing a strong argument that the allodynic response does involve microglial B_1_R. This is in accordance with the pro-nociceptive role of many other G-protein coupled-receptors expressed on microglia, including TLR2-4, P2Y_12_R, CCR2, CX_3_CR1 [58].

### Hyperalgesia mediated by the B_1_R

The activation of spinal cord tachykinin NK-1 receptor with SP caused a hyperalgesic response in the tail-flick test, which was associated with the release of glutamate and NO in control rats [42,59,60]. Whereas intrathecal administration of des-Arg^9^-BK, the selective B_1_R agonist, had no effect on the nociceptive threshold in control rats, it evoked a transient hyperalgesia in STZ-diabetic rat, which was likely mediated by the release of pro-inflammatory mediators (NO, SP and glutamate) based on pharmacological blockade of NOS, NK-1R and NMDAR [7]. The present study extended this observation by demonstrating that thermal hyperalgesia elicited by the B_1_R agonist in STZ-diabetic rats requires intact microglia. This is consistent with the effects of pro-nociceptive mediators such as NO [61], ROS [49], SP [62] and glutamate [63], which can be released by microglia.

The therapeutic effects of B_1_R antagonists and microglia inhibitors on allodynia and hyperalgesia are unlikely due to changes in blood glucose concentrations since microglia inhibitors and intrathecal B_1_R antagonist reversed pain neuropathy without affecting hyperglycemia in STZ-diabetic rats. Although SSR240612 reduced significantly hyperglycemia at 3 h post-gavage, rats remained diabetics (glycemia > 18 mmol/L). The mechanism of the transient anti-hyperglycemic effect of SSR240612 could be attributed to the inhibition of islet inflammation (insulitis) as suggested earlier in studies using [Leu^8^]des-Arg^9^-BK as B_1_R antagonist in STZ-diabetic mice [64,65].

## Conclusion

This study provides the first pharmacological evidence that spinal dorsal horn microglial B_1_R contributes to early pain neuropathy in a rat model of type 1 diabetes. Microglial B_1_R induction could be related to the production and release of spinal pro-inflammatory cytokines such as IL-1β and TNF-α. Hence, microglial B_1_R may represent a promising therapeutic target for the treatment of diabetic pain neuropathy.

## List of abbreviations

anti-Iba-1: Anti-Ionized calcium binding adapter molecule 1; aCSF: artificial cerebrospinal fluid; BK: bradykinin; BSA:  bovine serum albumin; CGRP:  calcitonin-gene related peptide; des-Arg^9^-BK: des-Arg^9^-bradykinin; IL-1β: interleukin-1 beta; B_1_R: kinin B_1 _receptor; Lys-BK: Lys-bradykinin; MAPK: mitogen-activated protein kinase; NO: nitric oxide; NOS: nitric oxide synthase; PBS: phosphate buffered saline; qRT-PCR: quantitative real-time PCR; ROS: reactive oxygen species; STZ: streptozotocin; SP: substance P; NF-kappa B: transcriptional nuclear factor kappa B; TRPV1: transient receptor potential vanilloid 1; TNF-alpha: tumor necrosis factor alpha

## Competing interests

The authors declare that they have no competing interests.

## Authors' contributions

ST designed the study, performed the experiments, analyzed the data, drafted and interpreted critically the manuscript. EC helped in allodynia and tail-flick test experiments. JPD performed the intrathecal implantations. RC conceived and wrote the final version with important intellectual contribution. All authors read and approved the final manuscript.
